# Intrinsic Disorder Is a Common Feature of Hub Proteins from Four Eukaryotic Interactomes

**DOI:** 10.1371/journal.pcbi.0020100

**Published:** 2006-08-04

**Authors:** Chad Haynes, Christopher J Oldfield, Fei Ji, Niels Klitgord, Michael E Cusick, Predrag Radivojac, Vladimir N Uversky, Marc Vidal, Lilia M Iakoucheva

**Affiliations:** 1 Laboratory of Statistical Genetics, The Rockefeller University, New York, New York, United States of America; 2 Center for Computational Biology and Bioinformatics, Indiana University School of Medicine, Indianapolis, Indiana, United States of America; 3 Center for Cancer Systems Biology and Department of Cancer Biology, Dana-Farber Cancer Institute, Boston, Massachusetts, United States of America; 4 Department of Genetics, Harvard Medical School, Boston, Massachusetts, United States of America; 5 Indiana University School of Informatics, Bloomington, Indiana, United States of America; 6 Institute for Biological Instrumentation, Russian Academy of Sciences, Moscow, Russia; University of Toronto, Canada

## Abstract

Recent proteome-wide screening approaches have provided a wealth of information about interacting proteins in various organisms. To test for a potential association between protein connectivity and the amount of predicted structural disorder, the disorder propensities of proteins with various numbers of interacting partners from four eukaryotic organisms *(Caenorhabditis elegans, Saccharomyces cerevisiae, Drosophila melanogaster,* and *Homo sapiens)* were investigated. The results of PONDR VL-XT disorder analysis show that for all four studied organisms, hub proteins, defined here as those that interact with ≥10 partners, are significantly more disordered than end proteins, defined here as those that interact with just one partner. The proportion of predicted disordered residues, the average disorder score, and the number of predicted disordered regions of various lengths were higher overall in hubs than in ends. A binary classification of hubs and ends into ordered and disordered subclasses using the consensus prediction method showed a significant enrichment of wholly disordered proteins and a significant depletion of wholly ordered proteins in hubs relative to ends in worm, fly, and human. The functional annotation of yeast hubs and ends using GO categories and the correlation of these annotations with disorder predictions demonstrate that proteins with regulation, transcription, and development annotations are enriched in disorder, whereas proteins with catalytic activity, transport, and membrane localization annotations are depleted in disorder. The results of this study demonstrate that intrinsic structural disorder is a distinctive and common characteristic of eukaryotic hub proteins, and that disorder may serve as a determinant of protein interactivity.

## Synopsis

From the formulation of Emil Fisher's lock-and-key hypothesis in 1894 until the early 1990s, a dominating and widely accepted concept in molecular biology was the protein structure–function paradigm. According to this concept, a protein can perform its biological function(s) only after folding into a specific rigid 3-D structure. Only recently has the validity of this structure–function paradigm been seriously challenged, primarily through the wealth of counterexamples that have gradually accumulated over the past 15 years. These counterexamples demonstrated that many proteins exist in a natively unfolded (or intrinsically disordered) state, and function without a prerequisite stably folded structure. In many cases, the lack of structure is required for biological function. Previous results have implicated intrinsic disorder as having an important role in protein interactions. The authors generalize this notion by comparing interaction networks from four eukaryotic organisms: yeast, worm, fly, and human. They have found that within these networks the proteins that interact with multiple protein partners (network hubs) are significantly more disordered than proteins that interact with a single protein partner (network ends). The results of this study demonstrate that intrinsic structural disorder is a distinctive and common characteristic of hub proteins, and that disorder may serve as a determinant of protein interactivity.

## Introduction

Systematic binary protein–protein interaction maps with various percentages of proteome coverage are currently available for S. cerevisiae [1,2], C. elegans [3], D. melanogaster [4], H. pylori [5], and, most recently, for H. sapiens [6,7]. As a result of these studies, it is now proposed that most networks within the cell have similar overall broad-scale topology where most proteins interact with just a few partners and a small number of proteins interact with many partners. Although all currently available networks represent only samples of the complete interactomes [8], the investigation of such partial networks is a first step toward a systems-biology understanding of cells and organisms.

While much has been learned to date about the general mechanisms of protein–protein interactions, the specific structural features that account for differences in protein interactivity are still unknown. It has recently been suggested that intrinsically disordered (ID) proteins play an important role in protein–protein interactions [9,10]. ID proteins and protein regions lack a unique 3-D structure and exist in a dynamic ensemble of conformations. More than 427 proteins containing 802 disordered regions have been annotated (http://www.disprot.org). Computational estimates suggest that eukaryotic proteomes have a significantly higher occurrence of ID proteins relative to prokaryotic proteomes [11,12]. The prevalence of ID proteins in eukaryotes is likely to be due to more complex signaling and regulatory pathways that heavily rely on disordered proteins [13]. Many ID proteins have been shown to mediate interactions through a disorder-to-order transition upon binding to their biological targets [14,15]. The lack of prior structure provides several advantages to ID-mediated protein interactions relative to interactions between folded proteins, such as decoupling of specificity and affinity, and the ability to recognize multiple binding partners with distinct interaction surfaces. In addition, the interaction interface areas of ID proteins is in general much larger per residue [16], which suggests that ID proteins would make more efficient hub proteins relative to ordered proteins [17].

Recent reviews [18–20] discuss the importance of intrinsic disorder for protein–protein interactions that involve binding to multiple partners. These reviews focus on individual examples of hub proteins with known disordered regions. However, no systematic study of organism-specific protein interaction networks that investigate their disorder content is currently available.

The hypothesis that intrinsic structural disorder may be an important attribute that can distinguish between hub and end proteins is tested in the present study. The prediction of disorder in the interaction networks from four eukaryotic organisms is carried out using PONDR VL-XT [21,22]. The comparison of proteins from these networks shows that while the disorder content varies between organisms, hub proteins are consistently found to be more disordered than end proteins in all organisms.

## Results

### Datasets Characterization

Protein interaction datasets from four eukaryotes, C. elegans (WORM), H. sapiens (HUMAN), D. melanogaster (FLY), and S. cerevisiae (YEAST) were selected for this study (Table 1). High-throughput datasets with experimentally demonstrated verification rates between 75% and 80% were selected for WORM and HUMAN; the literature-curated low-throughput dataset was selected for YEAST; and the literature-curated dataset that also included the high-throughput interactions was selected for FLY (Materials and Methods)*.* Although another FLY dataset consisting of only high-confidence high-throughput interactions also was available [4], it was not particularly useful because highly connected proteins (i.e*.*, hubs) were removed with the intent of reducing the number of nonspecific interactions. Subsequently, four additional datasets (WORM BioGRID, HUMAN HPRD, FLY BioGRID, and YEAST BioGRID) from two public databases [23,24], to which no confidence-based filtering have been applied (Materials and Methods, Table S1), were investigated for comparison.

**Table 1:**
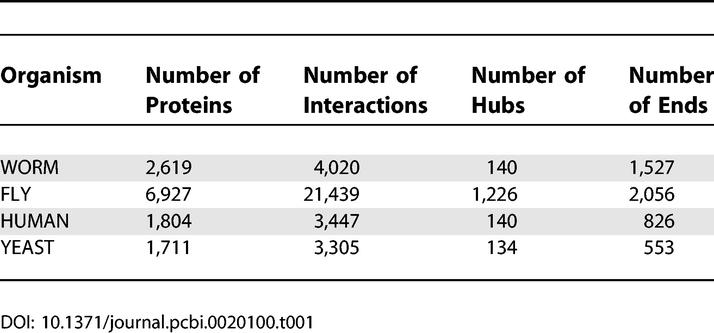
Properties of Protein Interaction Datasets from Four Organisms

From these datasets, ends and hubs were defined as proteins with one and ten or more interacting partners, respectively. Although this cutoff was chosen somewhat arbitrarily, the results of future analysis did not depend significantly on the cutoff value (unpublished data). The gap in the definition between hubs and ends was intended to buffer the classes, and should be considered as a conservative classification of hubs and ends. As shown in Table 1, the number of ends is between ~2-fold to 10-fold greater than the number of hubs, which is consistent with a scale-free network topology.

### Analysis of Disorder Predictions

**Figure 1:**
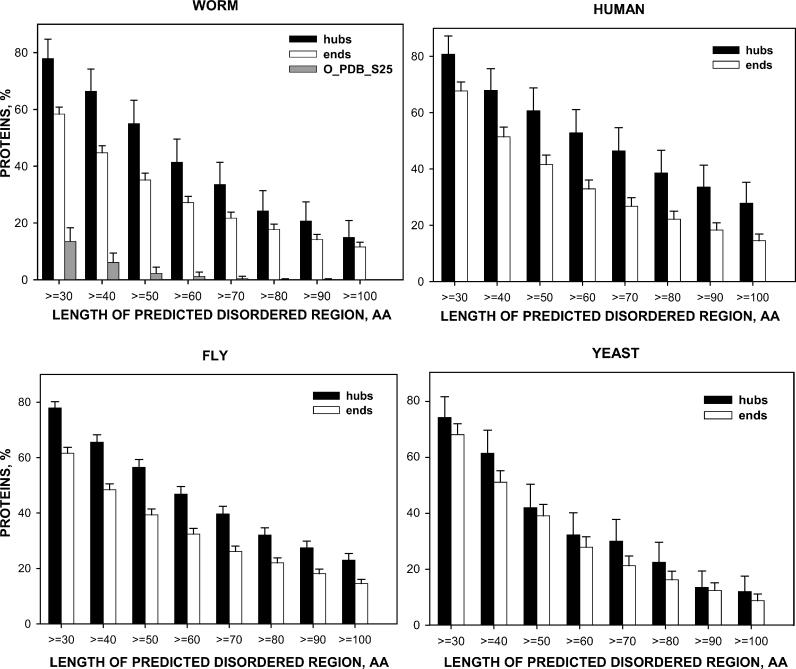
The Percentages of Hub and End Proteins with ≥30 to ≥100 Consecutive Residues Predicted to Be Disordered 95% confidence intervals were calculated using normal test for two binomial proportions.

Predictions of intrinsic structural disorder were carried out on four datasets using PONDR VL-XT [21,22]. As shown in Figure 1, significant differences between hubs and ends in the percentages of proteins containing predicted disordered regions of various lengths are observed. For example, 78% of hub proteins in WORM carry predicted disordered regions of ≥30 consecutive residues, whereas only 58% of end proteins have this characteristic. The prediction error rate of PONDR VL-XT (i.e., the prediction of disorder on the completely ordered dataset O_PDB_S25, see Materials and Methods) for this disorder length is ~13%, and it gradually decreases as the length of the predicted disordered region increases. The significant differences in the disorder content between WORM hubs and ends are observed for most disorder lengths, thereby indicating that WORM hub proteins are overall more disordered than WORM end proteins. Similar conclusions arise for two other organisms, HUMAN and FLY. In YEAST, however, significant differences in the percentages of proteins with predicted disordered regions are observed for only two disorder lengths (≥40 and ≥70). By comparison, the analysis of a much larger YEAST BioGRID dataset shows that the disorder content of hubs and ends is significant for all disorder lengths for this organism (Figure S1). In addition, the results of the disorder predictions for the remaining BioGRID and HPRD datasets (Table S1) are also consistent with a significantly greater amount of disorder in hubs relative to ends (Figure S1).

When hubs from all four organisms are compared with each other, HUMAN hubs have the overall highest disorder content (i.e*.*, higher percentage of proteins with predicted disordered regions) for all disorder lengths, whereas YEAST hubs have the lowest. Interestingly, when ends from all four organisms are compared with each other, HUMAN ends again have the highest disorder content. This suggests that the HUMAN interaction network has the highest disorder content among all studied organisms, in agreement with the predicted disorder content of the entire human proteome [12]. It should also be noted that disorder predictions for proteins with an intermediate number of partners (from 2 to 9) generally fall in between the predictions for hubs and ends (Figures S2 and S3).

**Figure 2:**
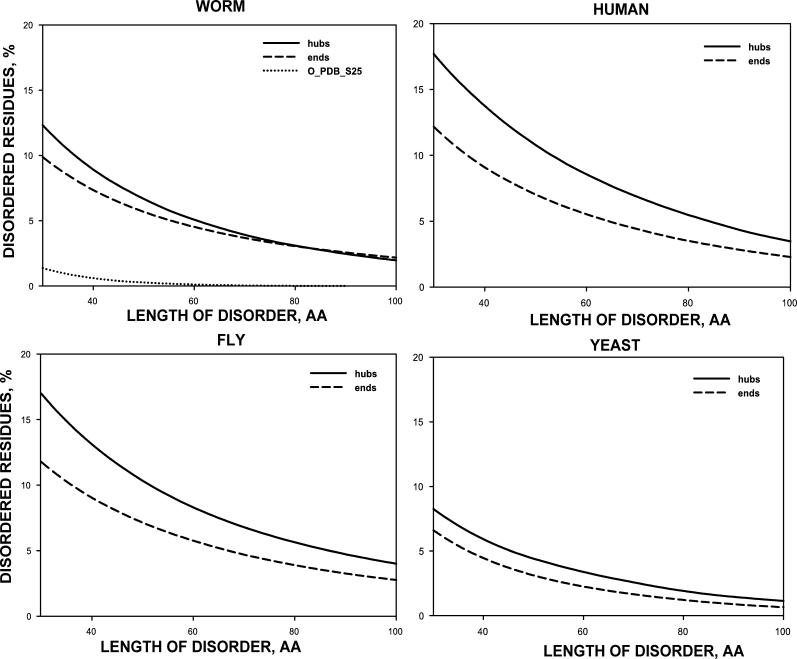
The Percentages of Residues Predicted to Be Disordered within Segments of Length ≥ the Value on the *x*-Axis

Since PONDR VL-XT predicts disorder on a per-residue basis, it is important to account for the differences in protein lengths when comparing predictions for entire datasets, because longer proteins are expected to have a greater number, as well as longer regions, of predicted disorder in comparison with shorter proteins. To compensate for the length dependency of disorder predictions, the per-residue disorder predictions were normalized by protein length. The percentages of disordered residues within segments of all possible lengths (starting from one and ending with the longest disordered region in the dataset) were calculated for all proteins, and then plotted against the predicted disordered region length (Figure 2). The same procedure was repeated using a completely ordered set of proteins (O_PDB_S25) to estimate the error rate of the predictions. The length-normalized predictions further confirm the differences in the disorder content of hubs and ends. The percentages of predicted disordered residues in hubs are generally higher than in ends (Figure 2), although the differences between hubs and ends are more apparent for the HUMAN and FLY than for the WORM and YEAST datasets. Furthermore, WORM hubs and ends have similar percentages of predicted disordered residues within long segments of disorder (80 residues and longer). When length-normalized predictions are considered, the proportion of predicted disordered residues is highest in the HUMAN dataset, and lowest in the YEAST dataset (Figure 2).

### Analysis of Various Disorder Parameters

To determine which specific disorder attributes contribute toward the differences observed between hubs and ends in each dataset, seven additional disorder parameters were calculated (see Materials and Methods for details). The results of a *t*-test for three representative disorder attributes *(RdisAA, avgScore,* and *RnumDR)* are shown in Table 2. The average disorder scores for hubs and ends were significantly different in all four organisms (*p* < 0.05). In addition, the relative number of predicted disordered residues was significantly (*p* < 0.005) different in WORM, HUMAN, and FLY. The difference between hubs and ends in the relative number of predicted disordered regions was significant (*p* < 0.01) for WORM and FLY. There were also significant differences in the relative number of short disordered regions for FLY (*p* < 0.05), medium disordered regions for WORM and FLY (*p* < 0.05), and long disordered regions for WORM, HUMAN, and FLY (*p* < 0.05) (Table S2). These data suggest that hubs have a higher disorder score in all four datasets, and a greater number of disordered residues and disordered regions in some datasets.

**Table 2:**
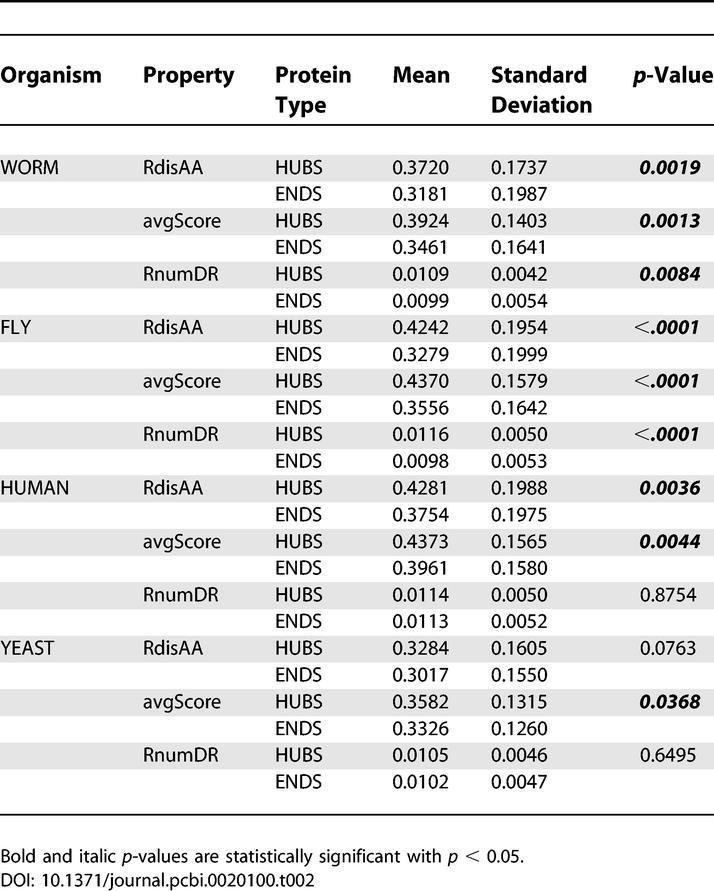
Representative Disorder Attributes Calculated for Four Datasets

### Consensus-Based Classification of Hubs and Ends

Cumulative distribution function (CDF) and charge-hydropathy (CH) consensus predictions were applied to the hubs and ends from four organisms and have been summarized in Table 3. Consensus predictions, as well as CDF and CH predictions, assume that all proteins can be classified as wholly ordered or wholly disordered. While this assumption is certainly an oversimplification for proteins containing both ordered and disordered regions, these predictions can be interpreted as representative of the predominant order/disorder composition of a protein. Unclassified proteins do not necessarily consist of an equal number of ordered and disordered residues, but rather are proteins whose sequences give conflicting indications of their order/disorder composition by two different methods (Materials and Methods). The view of disorder in these networks provided by consensus predictions (Table 3) represents an alternative analysis to residue-based predictions (Figures 1 and 2).

**Table 3:**
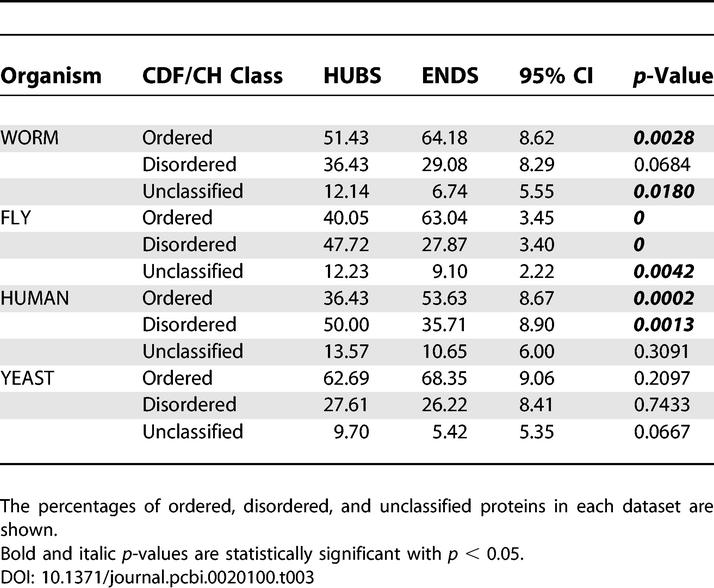
Results of a Binary Classification Using Consensus Method

The results indicate an enrichment of wholly disordered proteins and a depletion of wholly ordered proteins in hubs, relative to ends, which is consistent with the analysis of residue-based predictions. HUMAN and FLY hubs are significantly enriched in wholly disordered proteins compared with ends (*p* < 0.005). WORM and YEAST hubs are similarly enriched, but the differences between hubs and ends are found to be only borderline significant for WORM, and insignificant for YEAST. It should be noted that when the larger YEAST BioGRID dataset is used, the data for YEAST also becomes significant (Table S3). Conversely, hubs are depleted in wholly ordered proteins, relative to ends. This difference is statistically significant for the WORM, HUMAN, and FLY datasets, as well as for the YEAST BioGRID dataset (Table S3). Also, all datasets show a higher proportion of unclassified proteins in hubs, relative to ends, where these differences are significant (*p* < 0.05) for the WORM and FLY datasets. It has previously been suggested that disagreement between CDF and CH may have important structural and functional implications [25], although this remains to be tested experimentally.

YEAST hub proteins demonstrate a bias toward enrichment in wholly disordered proteins and depletion in wholly ordered proteins, but these differences are statistically insignificant for the smaller, literature-curated YEAST dataset (Table 3), and they are significant for the larger YEAST BioGRID dataset (Table S3). This suggests that an association of hubs with disorder may be weaker in YEAST than in other studied organisms, because it could only be detected when a larger dataset is used. A potential explanation is that yeast is a unicellular organism while the others are multicellular organisms, and the requirements for intracellular regulation are likely very different in unicellular organisms than in multicellular organisms, with this difference reflected in the lower bias of YEAST hubs toward disorder.

### Analysis of the Amino Acid Composition of Ordered and Disordered Hubs and Ends

Ordered and disordered proteins and protein regions have significantly different amino acid compositions [26]. Disordered proteins are characterized by enrichment in hydrophilic, charged, and structure-breaking amino acids and by depletion in hydrophobic and aromatic amino acids as compared with the ordered proteins.

**Figure 3:**
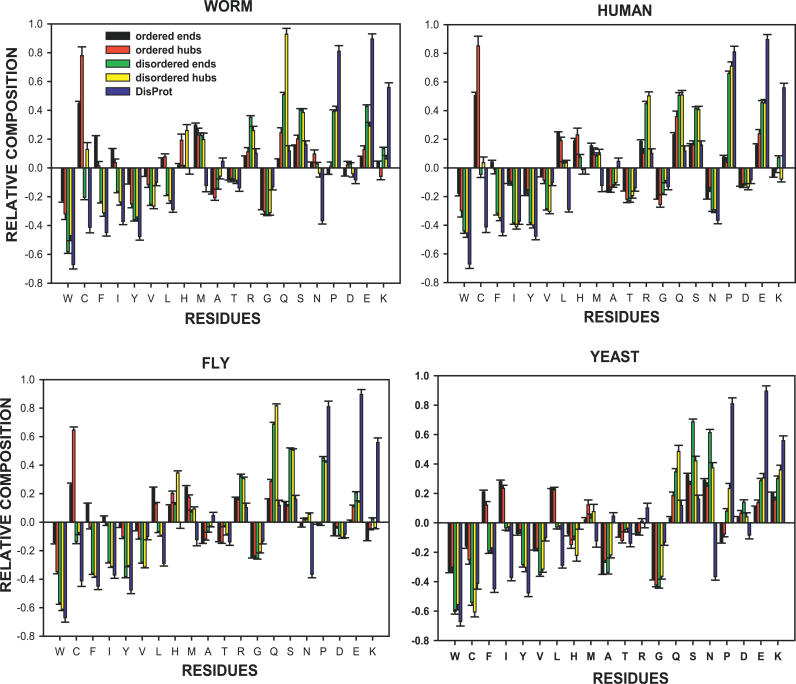
Amino Acid Compositions of Hub, End, and Disordered Proteins Are Shown Relative to the Composition of Completely Ordered Globular Proteins Amino acids are arranged from left to right in order of increasing flexibility as defined by Vihinen et al. [52]. The error bars were calculated as explained in Materials and Methods.

Here, amino acid compositions of ordered and disordered hubs and ends were compared with the amino acid composition of disordered protein regions extracted from the DisProt database [27] (Figure 3). The composition of each dataset is plotted relative to the composition of a subset of well-ordered globular proteins extracted from the PDB Select 25 database [28]. The negative value of the bar therefore signifies that the dataset is depleted in a particular amino acid residue in comparison with the ordered proteins while the positive value signifies enrichment.

A striking and common feature of three datasets (WORM, HUMAN, and FLY) is an extreme enrichment of ordered hubs (and, to a lesser degree, ordered ends) in cysteine. Since cysteines are frequently involved in disulphide bonds that usually serve to stabilize the protein structure, it is not surprising to find the enrichment of the ordered proteins in cysteine. Besides their role in disulphide bond formation, cysteines are also common in Zn finger domains that are often found in the nucleic acid–binding proteins. This may suggest that ordered hubs and ends are enriched in proteins that also bind to DNA and RNA (in addition to binding to other proteins). Interestingly, ordered YEAST hubs and ends as well as disordered YEAST hubs and ends all are depleted in cysteine, similar to DisProt proteins (Figure 3). Another interesting feature observed in WORM and FLY (and to a smaller extent in HUMAN and YEAST) is an extreme enrichment of disordered hubs and ends in glutamine. Since glutamine is usually found on protein surfaces and its polar side-chain is often involved in protein active or binding sites, it is not unexpected to observe the enrichment of hubs in this residue.

By similarity with other disordered proteins, disordered hubs and ends are generally depleted in aromatic and aliphatic amino acids in all organisms (see the left part of Figure 3), and are enriched in proline and serine. At the same time, disordered YEAST hubs are enriched in asparagine, unlike DisProt proteins.

Thus, the amino acid composition analysis shows that the large compositional biases are observed between the ordered and the disordered subclasses of hubs and ends in all studied organisms. Another observation is that YEAST has more distinctive compositional biases than the other three organisms.

### The Analysis of GO Annotations

**Figure 4:**
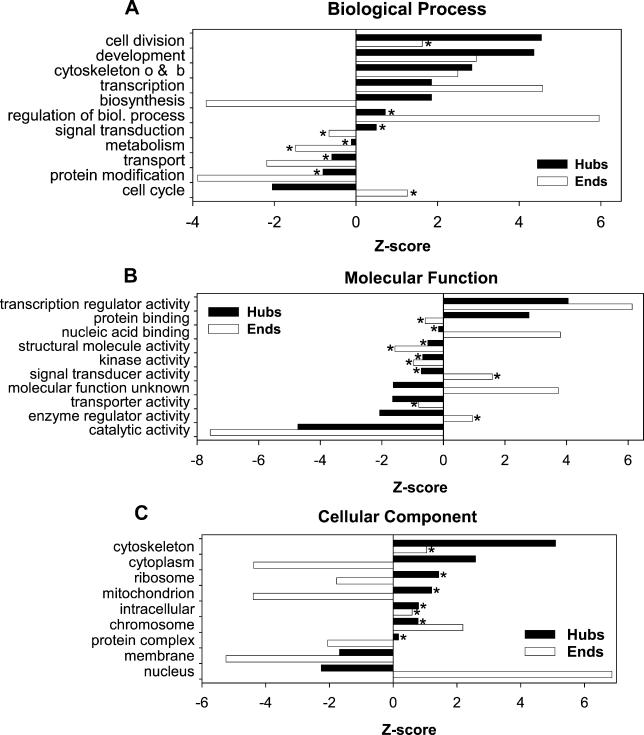
Association of PONDR VL-XT Predicted Disorder with GO Annotations in Yeast A positive (negative) *Z*-score indicates that more (less) disorder is associated with the indicated annotation than would be expected by chance. The null distributions for hubs (black bars) and ends (white bars) were generated separately. All associations are significant at a type I, test-wise error rate of 0.05, unless indicated by asterisks. The three branches of GO annotations are plotted separately: (A) biological process, (B) molecular function, and (C) cellular component. o, organization; b, biogenesis.

GO annotations for yeast have the longest history of any of the GO annotated organisms [29], and are generally considered to have the highest quality and completeness. For this reason, we selected yeast to examine the relationship between GO annotations and order and disorder in hubs and ends. The null model distribution was generated by a permutation test (Materials and Methods). The *Z*-score for the observed number of disordered residues associated with each selected high-level annotation is plotted in Figure 4.

Difficulties with multiple testing correction and correlated annotations have been identified as a weakness of many approaches to the proteome-wide functional analysis [30]. However, these factors do not play a significant role in this functional study. Multiple testing was not corrected for since GO annotations of interest were selected a priori, resulting in far fewer tests than in previous analyses of this kind [12,31]. Correlation between parameters is handled implicitly through the randomization procedure and does not have a significant effect on the results. Caution should be used in interpreting these results with regard to the classic correlation versus causation problem. For example, positive associations do not necessarily imply that the annotation is responsible for a given function, only that proteins with that function are enriched in disordered residues.

Disorder is found to be enriched in both hubs and ends with several annotations from the biological process ontology (Figure 4A), including development, cytoskeleton organization and biogenesis, and transcription. Hubs and ends involved in cell division processes also are enriched in disorder. The enrichment of disorder for these functions is consistent with the hypothesis that disorder is highly involved in functions specific to eukaryotes, relative to the other kingdoms [11,12]. Hubs are only significantly depleted for one process annotation, the cell cycle, whereas the ends involved in the cell cycle are enriched in disorder. Interestingly, hubs involved in biosynthesis are enriched in disorder, but ends with the same annotation are highly depleted in disorder. Finally, ends with an annotation “regulation of biological processes” show a large enrichment in disorder while hubs show no significant bias. Note that this GO term is very broad, covering all types of biological processes.

Only one of the molecular function GO annotations examined here, the “transcription regulator activity” (Figure 4B), is associated with disorder in both hubs and ends. The previously noted strong disorder bias of proteins involved in transcription regulation [32] agrees well with these results. Hubs involved in protein binding also are strongly associated with predicted disorder, which agrees with the hypothesis that disorder is a common mediator of protein–protein interactions [18]. Hubs with molecular transport, enzyme regulation, and catalytic activity annotations are found to be depleted in predicted disorder, in support of previous observations of low disorder content in proteins involved in catalytic cellular functions [13].

Significant biases for ends are similar to the biases of hubs, except for “nucleic acid–binding activity,” for which ends show a large enrichment in disorder. DNA-binding domains are frequently well-ordered [32], whereas RNA-binding proteins can be completely disordered (such as ribosomal proteins when separated from the ribosome) [15], or they can carry long disordered domains (such as SR splicing factors) [33]. The nucleic acid–binding annotation was also found to be associated with disorder in another study [12].

Lastly, analysis of the cellular component ontology (Figure 4C) gives several interesting results. Cytoplasmic hubs are biased toward disorder whereas nuclear hubs are biased toward order. Ends, on the other hand, show the opposite biases. Previous studies have found that nuclear proteins are typically enriched in disorder while cytoplasmic proteins are typically depleted in disorder [12], which agrees with the results for ends. The enrichment of ends localized to the chromosome in disorder agrees with the enrichment of disorder in ends that bind nucleic acids. Finally, hubs localized to the cytoskeleton are highly enriched in disorder. Interestingly, the high disorder content of the cytoskeletal proteins that is comparable to the disorder content of regulatory and cell signaling proteins has previously been observed [13]. In addition, disorder has been shown to be crucial in the function of at least one structural molecule, the bacterial flagellar protein FlgM [34]. The disorder in cytoskeletal proteins is examined in more detail below.

### Examination of Specific Hub Examples

To correlate the disorder predictions with available structural information, all yeast hubs with cytoskeleton localization GO annotation were examined (Table 4). Cytoskeletal proteins are predicted to contain a large amount of disorder [13], therefore it seems beneficial to examine them in detail. As shown in Table 4, limited structural information is available for these proteins. Only for one protein, Act1p, out of fifteen has the structure of the full-length protein been determined, whereas for the remaining four proteins the structures are known only for portions of the total sequences. The total percentage of residues in these five proteins, for which the structural information is available, constitutes only 11.3%.

**Table 4:**
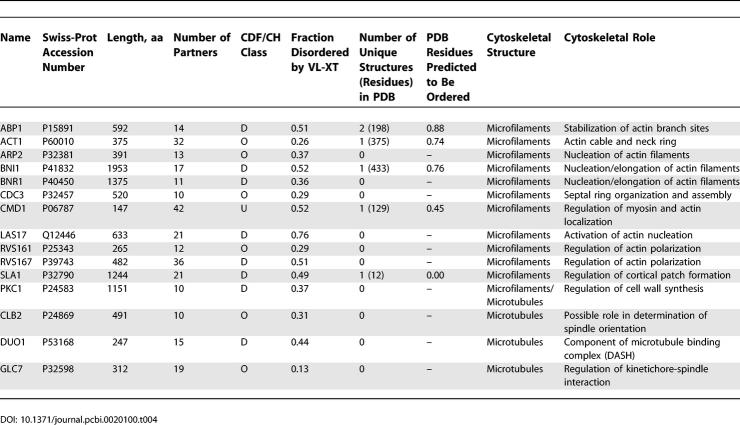
Structural and Functional Features of Yeast Cytoskeletal Hubs

Comparison of the structured regions to disorder predictions shows good agreement for most structures, i.e., regions with known structures are predicted to be highly ordered, with two exceptions, Cmd1p and Sla1p. In the case of Sla1p, an 11-residue segment of this protein is bound to a Src homology (SH3) domain (PDB code 1SSH). Classical partners of SH3 domains are rich in proline residues, and this region of Sla1p contains five proline residues. Proline is a strong promoter of intrinsic disorder [26], and it is likely that this region is intrinsically disordered (as correctly predicted by PONDR VL-XT), but it undergoes a disorder-to-order transition upon binding to SH3 domain. In the case of Cmd1p (calmodulin), the solution structure shows a high degree of flexibility (PDB code 1LKJ). The N-terminal and C-terminal EF-hand domains cannot be aligned simultaneously in most of the model structures due to a highly dynamic central linker. A large degree of flexibility is also apparent at both termini of Cmd1p. These three regions (both termini and a linker between EF-hand domains) are predicted to be disordered (in agreement with the high degree of flexibility in the NMR structure), with much of the EF-hand domains predicted to be ordered (unpublished data). Therefore, the disorder predictions generally agree with the available structural information.

**Figure 5:**
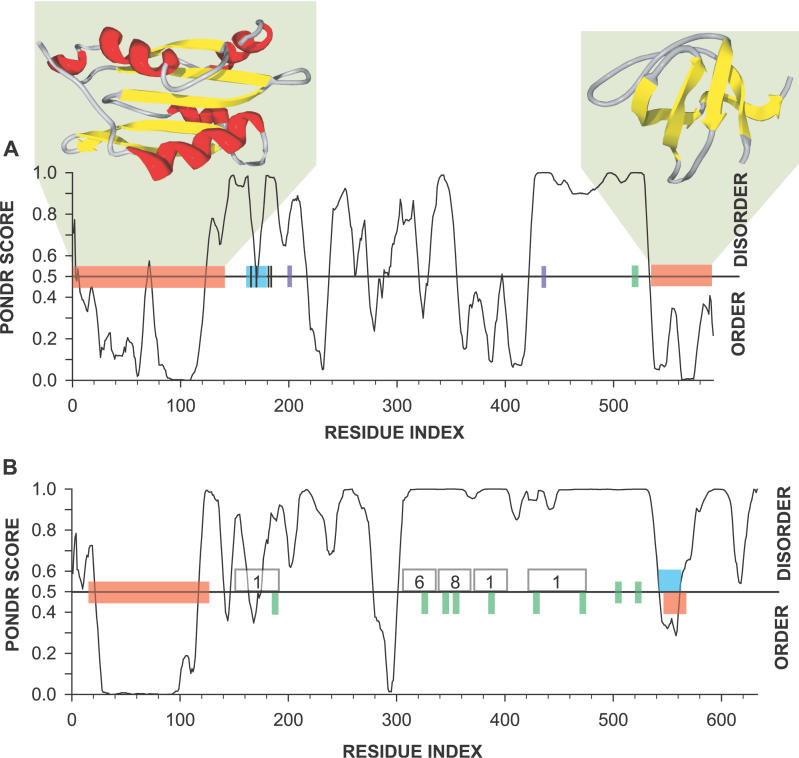
PONDRing Abp1p and Las17p (A) The PONDR VL-XT prediction for Abp1p is plotted along with bars representing the positions of the ADF domain (N-terminal orange bar, structure 1HQZ), the SH3 domain (C-terminal orange bar, structure 1JO8), a poly-proline region (green bar), a predicted α-MoRF (blue bar), known phosphorylation sites (black hash marks), and regions critical for Arp2/3 activation (purple bars). (B) The PONDR VL-XT prediction for Las17p is plotted along with bars representing the positions of the WASP homology domain 1 (N-terminal orange bar), WASP homology domain 2 (C-terminal orange bar), poly-proline regions (green bars), and a predicted α-MoRF (blue bar). The number of interaction partners associated with a given region [39] is indicated in the numbered boxes.

The functional role that disorder may play in cytoskeletal hubs was further investigated by comparing PONDR VL-XT predictions of two disordered hubs, Abp1p and Las17p, to known features of these proteins (Figure 5). These two proteins are selected as examples because they are both well-studied and predicted to be highly disordered.

Actin-binding protein 1 (Abp1p) was the first actin-binding protein identified in yeast [35]. This protein is predicted to be highly disordered (Table 4), but has two regions of known structure that correspond to regions predicted to be ordered (Figure 5A). The N-terminal actin depolymerization factor-homology (ADF-H) domain is the primary factor responsible for actin binding. The C-terminal SH3 domain binds to nonclassical proline motifs. Abp1p also serves as a target for at least one SH3 domain; the SH3 domain of Rvs167p binds to the C-terminal proline-rich region (Figure 5A, green bar), which is predicted to be disordered.

Abp1p contains other protein-binding features associated with predicted disorder. For example, its two central acidic domains mediate interaction with the Arp2/3 complex [36]. Two sets of contiguous triplet mutations in these domains (Figure 5A, purple bars), both separately and combined, completely disrupt the interaction of Abp1p with the Arp2/3 complex and the associated activation of the complex [36]. Finally, Abp1p contains a predicted α-molecular recognition feature [31] (α-MoRF, Figure 5A, blue bar). These features are responsible for mediating protein interaction through a disorder-to-order transition upon binding to partners. Interestingly, this prediction coincides with four observed serine/threonine phosphorylation sites [37]. The identities of the kinases that phosphorylate these sites have not yet been established.

Las17p is a yeast homolog of the Wiskott-Aldrich Syndrome protein (WASP). Like Abp1p, Las17p is highly disordered (Table 4) and contains several regions that mediate protein interactions (Figure 5B). The WASp-homology domain 1 (WH1), located at the N-terminus of Las17p (Figure 5B, N-terminal orange box), is similar to SH3 and WW domains in that they mediate interactions with short sequence motifs. The 3-D structures of WH1 domains from other proteins with (e.g., PDB code 1DDV) and without (e.g*.*, PDB code 1DDW) binding partners have been determined. The WH2 domain at the C-terminus (Figure 5B, C-terminal orange box) of Las17p is a short, conserved, α-helical motif that mediates interaction with actin [38]. Interestingly, an α-MoRF is predicted to coincide with the WH2 motif (Figure 5B, blue box and C-terminal orange box, respectively), indicating that this WH2 motif likely functions through a disorder-to-order transition upon binding to actin. Solution of actin-WH2 complex structures from other proteins confirms that the WH2 domain forms an α-helix on binding actin (e.g., PDB code 2A41). The proline-rich regions of Las17p (Figure 5B, green boxes) have been shown to bind at least 17 distinct SH3 domain-containing protein partners [39] (Figure 5B, numbered boxes). All of these proline-rich binding regions are located within predicted disordered regions.

## Discussion

The investigation of the disorder content of proteins from four eukaryotic interactomes shows that hub proteins are more disordered than end proteins in all four studied organisms (Figures 1 and 2), even though the predicted disorder content differs among these organisms [11,12]. It is established that the proportion of disordered proteins correlates with the complexity of an organism [11,12]. The application of two different disorder predictors to proteins from the three kingdoms of life has shown that the disorder content of bacteria and archaea is significantly lower than that of eukaryotes. The amount of predicted disorder also varies among eukaryotes. Comparison of disorder predictions in complete eukaryotic genomes [11,12] shows that even though two different predictors were used (PONDR VL-XT [11] versus DISOPRED2 [12]), the prediction results agree in terms of the disorder content ranking, i.e., “Fly > Yeast > Worm” [11] (note that human genome was not available at that time), and “Human > Fly > Yeast > Worm” [12]. Interestingly, when the prediction of disorder was carried out on all proteins (hubs, ends, and proteins with two to nine partners) from the networks in the present study (unpublished data), the ranking “Human > Fly > Yeast > Worm” agreed with the previous studies that were carried out on complete genomes. At the same time, the relative percentages of predicted disorder in the networks were generally higher than those reported previously for the complete genomes [11], even though the same predictor PONDR VL-XT was used in both studies. This result may indicate that proteins that interact with other proteins are on average more disordered than proteins that interact with ligands, such as nucleic acids, small molecules, lipids, etc.

Another interesting observation that follows from comparison of the networks to the complete genomes is that the disorder content of the proteomes is closer to the disorder content of ends than to the disorder content of hubs (unpublished data). Although differing views regarding the scale-free nature of the protein interaction networks exist [40,41], it is still tempting to speculate that this bias could be explained by a potentially higher fraction of ends as compared with hubs in all genomes.

We previously determined that human cell signaling and cancer-associated proteins are significantly more disordered than proteins from other functional categories [13]. Interestingly, the disorder content of HUMAN hubs (Figure 1) is very similar to that of human regulatory and cancer-associated proteins, suggesting that many cell signaling and regulatory proteins are network hubs.

The high disorder content of hubs relates directly to their function. Intrinsic disorder provides several important functional benefits for interactions with multiple partners. First, it allows hubs to adapt to the structure of a variety of differently shaped binding partners. Such structural malleability is especially important for hubs that interact with their partners using the same or overlapping binding surfaces. Second, disorder may enable a hub protein to elicit both inhibiting and activating effects on different partners, as was recently noted for moonlighting proteins [42]. Third, structural plasticity may enable some proteins to serve as hubs in multiple and distinct signaling networks. One example of such a hub is glycogen synthase kinase 3β, which uses two different ID regions to participate in two unrelated signaling pathways, Wnt and insulin signaling [18].

While intrinsic disorder is an important feature of hub proteins, many ordered hub proteins also exist [18]. Interestingly, it has been recently proposed that ordered hubs have higher surface charge than nonhub proteins, and that this increased charge is likely to have an impact on their binding ability [43]. Furthermore, it has been noted that the binding partners of several ordered hubs are intrinsically disordered [18]. The examples include the partners of 14-3-3 proteins [44] the partners of β-catenin [45], and the partners of several other proteins (such as calmodulin, actin, and Cdk) [18]. The results of the present study suggest that wholly ordered hubs, as defined by the CDF/CH consensus classification, constitute a substantial fraction of all hub proteins and are especially prevalent in the YEAST dataset (Table 3).

Among all the networks examined here, the YEAST interaction network appears to exhibit the smallest difference between hubs and ends in terms of predicted disorder, at least when literature-curated interactions are considered (Figure 1, Tables 2 and 3, compare with Figures S1 and S3). Notably, the amino acid composition of proteins from the YEAST network appears to be the least similar to the three other organisms (Figure 3). In addition, the proportion of wholly ordered proteins within both YEAST hubs and YEAST ends is the highest among the four datasets (Table 3, Table S3). A plausible explanation of the smaller differences in disorder content of YEAST hubs and ends is that the interactomes of the unicellular organisms are inherently simpler than metazoan interactomes due to less sophisticated signaling and regulation pathways. Because of their greater simplicity, these yeast pathways may rely less heavily on disorder than the networks of higher eukaryotes.

In summary, the present study shows that intrinsic structural disorder is a distinctive and common characteristic of eukaryotic hub proteins, and it suggests that disorder may serve as a determinant of protein interactivity. In the future, it would be interesting to compare more specialized signaling and metabolic networks to each other to determine whether the high disorder content of hubs is a common feature of all cellular networks. In addition, it would certainly be interesting to perform the disorder analysis on the complete interactomes (when they are available) to determine whether similar conclusions are reached.

## Materials and Methods

### Datasets.

The protein–protein interaction datasets for each organism (Table 1) were constructed as follows: (i) The interaction dataset for C. elegans (WORM) corresponds to the “First-Pass” interactions of the worm interactome version 5, or “WI5” [3]; (ii) The interaction dataset for H. sapiens (HUMAN) represents a union of the CCSB human interactome version 1, or “CCSB-HI1” extracted from Rual et al. [6] and high-confidence interactions with three or more quality points extracted from Stelzl et al. [7]; (iii) The interaction dataset for D. melanogaster (FLY) represents a union of literature-curated *Drosophila* interactions stored in the BIND (http://www.bind.ca), DIP (http://dip.doe-mbi.ucla.edu), and MINT (http://mint.bio.uniroma2.it/mint) interactions databases; (iv) The interaction dataset for S. cerevisiae (YEAST) represents the union of literature-curated yeast interactions stored in the BIND, DIP, and MINT interactions databases; (v) The dataset O_PDB_S25 contains only ordered parts of proteins extracted from the database PDB Select 25 [28]. The disorder predictions on this mostly nonredundant dataset served as a control for estimating the false-positive prediction error rate; (vi) DisProt dataset consists of experimentally verified disordered protein regions extracted from the DisProt database [27]. Four additional datasets, WORM BioGRID, HUMAN HPRD, FLY BioGRID, and YEAST BioGRID (Table S1), to which no confidence-based filtering have been applied, were extracted from BioGRID [23] and HPRD [24] and used for comparison.

The redundancy removal from all datasets did not significantly reduce the number of interactions. On average, only 2.2% of interactions were removed at 70% protein sequence identity level, and 15.6% of interactions were removed at 30% protein sequence identity level (unpublished data). Therefore, the original datasets were used in the present study.

Since a clear definition of a hub protein, in terms of a number of interacting partners, is not well-established, and since the definition might vary from one dataset to another, we somewhat arbitrarily chose ten partners as a cutoff value and defined proteins with ≥10 partners as *hubs*. Proteins with one interacting partner are defined here as *ends*. However, it should be mentioned that varying the cutoffs of hub definition gives rise to similar results (Figures S2 and S3).

### Disorder predictions.

Predictions of intrinsic disorder were carried out using a well-characterized disorder predictor PONDR VL-XT [21,22]. This predictor was trained on the experimentally (X-ray and NMR) confirmed disordered protein regions, while the ordered training set included completely ordered proteins extracted from the nonredundant set of proteins from PDB Select 25 [28]. The accuracy of this predictor, benchmarked on the 42 CASP5 targets, reached 72.8% [46]. PONDR VL-XT is currently being used successfully to guide the removal of disordered regions that interfere with crystallization of problematic proteins for high-throughput structure determination [47]. Access to PONDR VL-XT (http://www.pondr.com) was provided by Molecular Kinetics (Indianapolis, Indiana, United States).

### Disorder parameters.

The following disorder parameters (Table 2) have been calculated for all studied datasets: (i) *disAA,* the number of predicted disordered residues in the protein; (ii) *avgScore,* the average disorder prediction score for an entire protein; (iii) *shortDR,* the number of continuous, predicted disordered regions of length 10–30 amino acids; (iv) *medDR,* the number of continuous, predicted disordered regions of length 31–60 amino acids; (v) *longDR,* the number of continuous, predicted disordered regions of length 61–longest DR; (vi) *numDR,* the number of continuous, predicted disordered regions of length 10–longest DR; (vii) *maxDR,* the longest predicted disordered region in the protein. To eliminate the dependency of calculated parameters on protein length, the relative values of the attributes *(RdisAA, RshortDR, RmedDR, RlongDR, RallDR,* and *RmaxDR)* were derived by dividing the numerical value of each attribute by the protein length. Student's *t*-test was used to calculate *p*-values in Table 2.

### Consensus classification.

Predictions of wholly ordered and wholly disordered proteins (Table 3) were made as previously described [25]. Briefly, these predictions assume that proteins fall into one of two classes: wholly disordered or wholly ordered. PONDR VL-XT CDF classification [11] and CH classification [48] were used to make predictions based on the consensus between the two methods. A degree of confidence was derived for both methods, and, for the purposes of consensus prediction, predictions were taken as being either high or low confidence. If both methods agree, a protein is assigned to that class. If one method gives a high confidence prediction and the other a low confidence prediction, a protein is assigned the class indicated by the high confidence prediction. Finally, if the methods disagree and both give either high confidence or low confidence prediction, the protein is left unclassified. The normal test for two binomial proportions was used to calculate 95% confidence intervals and *p*-values for Table 3.

### Amino acid composition.

The amino acid composition analysis was performed as previously described [22]. Briefly, the mole fraction of the amino acid in a database was calculated as:

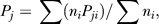
where *P_ji_* is the frequency of amino acid *j* in sequence *i* of length *n_i_*. The variances of the amino acids in the dataset were calculated as:

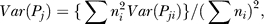
where *Var*(*P_ji_*) = *P_ji_*(1 *– P_ji_*)*/n_i_*.

The fractional difference in composition between two datasets *a* and *b* was calculated as 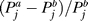 . The variances for these ratios were calculated as:

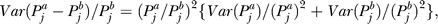
where 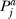 is the mole fraction of amino acid *j* in the dataset *a,* and 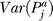 is the variance of amino acid *j* in the dataset *a*.

### GO annotations.

Gene Ontology (GO) [49] annotations for S. cerevisiae [29] were obtained from the GOA database [50]. The correlation between PONDR VL-XT disorder predictions and process/function/localization GO annotations were determined using an approach related to Fisher's permutation test [51]. This approach has been previously used to examine the association of disorder predictions and GO annotations [12]. In this test, a null distribution, which assumes no association between disorder predictions and annotations, is generated. Disorder predictions for adjacent residues are highly correlated due to overlapping compositional windows. To partially account for this, the observed disordered regions (rather than individual residue predictions) were permuted.

Predicted disordered regions were randomly distributed 10,000 times for hubs and ends separately, and the number of disordered residues associated with specific annotations was counted. This null distribution was used to calculate a *Z*-score for the observed counts for each annotation, and significance was evaluated based on the number of trials that contradicted the hypothesis indicated by the *Z*-score. The calculated *p*-values have not been corrected for multiple testing. High-level GO annotations of interest were selected prior to testing, and results were restricted to annotations with at least five examples in each of the hubs and ends sets.

## Supporting Information

Figure S1 The Percentages of Hub and End Proteins from BioGRID and HPRD with ≥30 to ≥100 Consecutive Residues Predicted to Be Disordered 95% confidence intervals were calculated using normal test for two binomial proportions.
(911 KB EPS)

Figure S2 The Percentages of All Interacting Proteins from Four Datasets with ≥30 to ≥100 Consecutive Residues Predicted to Be Disordered 95% confidence intervals were calculated using normal test for two binomial proportions.
(982 KB EPS)

Figure S3 The Percentages of All Interacting Proteins from BioGRID and HPRD with ≥30 to ≥100 Consecutive Residues Predicted to Be Disordered 95% confidence intervals were calculated using normal test for two binomial proportions.
(913 KB EPS)

Table S1 Properties of Protein Interaction Datasets Derived from BioGRID and HPRD (19 KB XLS)

Table S2 Disorder Attributes Calculated for Four Datasets (23 KB XLS)

Table S3 Results of a Binary Classification Using Consensus Method on BioGRID and HPRD Datasets The percentages of ordered, disordered, and unclassified proteins in each dataset are shown.
(19 KB XLS)

### Accession Numbers

Swiss-Prot (http://www.ebi.ac.uk/swissprot) accession numbers for proteins mentioned in this paper are: Abp1p (P15891), Act1p (P60010), Arp2 and Arp3 (P32381, P47117), Cmd1p (P06787), FlgM (P26477), Las17p (Q12446), Rvs167p (P39743), and Sla1p (P32790).

## Abbreviations

CDF: cumulative distribution function
CH: charge-hydropathy
WASP: Wiskott-Aldrich syndrome protein
